# Prevalence of obstructive sleep apnea syndrome in patients with pulmonary embolism: a prospective observational study

**DOI:** 10.1186/s12959-026-00895-0

**Published:** 2026-07-18

**Authors:** Mustafa Canbaz, Zeynep Zeren Uçar, Işıl Karasu, Dursun Tatar, Ahmet Emin Erbaycu

**Affiliations:** 1 Department of Chest Diseases, İzmir Dr. Suat Seren Chest Diseases and Thoracic Surgery, Training and Research Hospital, University of Health Sciences, İzmir, Türkiye Turkey; 2 Department of Sleep Disorders, İzmir Dr. Suat Seren Chest Diseases and Thoracic Surgery, Training and Research Hospital, University of Health Sciences, İzmir, Türkiye Turkey

**Keywords:** Obstructive sleep apnea syndrome, Pulmonary embolism, Apnea–hypopnea index, Nocturnal hypoxemia, Venous thromboembolism, WatchPAT

## Abstract

**Background:**

The relationship between obstructive sleep apnea syndrome (OSAS) and pulmonary embolism (PE) remains inadequately characterized, and prospective data obtained with objective sleep testing in confirmed PE patients are scarce. We aimed to determine the prevalence of OSAS in patients with radiologically confirmed PE and to compare clinical, laboratory, and polysomnographic features according to OSAS status.

**Methods:**

In this single-center prospective observational study, 45 patients with confirmed PE (thoracic computed tomography pulmonary angiography or ventilation/perfusion scintigraphy) underwent portable polysomnography (WatchPAT 200^®^) within two weeks of diagnosis. OSAS was defined as an apnea–hypopnea index (AHI) ≥ 5 events/hour. Group comparisons used the Mann–Whitney U test and chi-square or Fisher’s exact test; associations with AHI were assessed by Spearman’s rank correlation.

**Results:**

OSAS was identified in 31 patients (68.9%): 11 mild, 12 moderate, and 8 severe. Body mass index (BMI) was significantly higher in OSAS-positive patients [median 31.2 (IQR 26.5–34.6) vs. 27.3 (24.2–30.4) kg/m²; *p* = 0.037]. Blood urea nitrogen was also elevated in the OSAS group [36.0 (25.1–52.0) vs. 19.5 (16.2–43.5) mg/dL; *p* = 0.026]. Minimum nocturnal oxygen saturation was markedly lower in OSAS patients [83.0% (79.0–86.5) vs. 91.0% (88.5–92.0); *p* < 0.001], as were the oxygen desaturation index and time with SpO₂ <90% (both *p* < 0.001). AHI correlated positively with age (*r* = 0.363; *p* = 0.014) and negatively with minimum nocturnal SpO₂ (*r* =− 0.753; *p* < 0.001). Classical Wells criteria did not differ between groups; 38.7% of OSAS-positive patients had no identifiable Wells risk factor for PE.

**Conclusions:**

OSAS was present in nearly 70% of PE patients and was associated with greater nocturnal hypoxemic burden, higher BMI, and elevated blood urea nitrogen. The absence of classical precipitating factors in a substantial proportion of OSAS-positive patients is consistent with the hypothesis that sleep-disordered breathing may be associated with an increased thrombotic risk. Further prospective studies are needed to evaluate the clinical utility and cost-effectiveness of targeted OSAS screening strategies in PE patients, particularly those without conventional risk factors.

## Introduction

Obstructive sleep apnea syndrome is one of the most common, yet persistently underdiagnosed, sleep disorders in adults worldwide. Its hallmark — recurrent upper airway collapse during sleep — generates a cyclical pattern of intermittent hypoxia, arousal, and renewed ventilation that, over years, imposes a substantial physiological burden on the cardiovascular and hemostatic systems [1, 2]. The cardiovascular consequences of untreated OSAS, including hypertension, atrial fibrillation, and accelerated atherosclerosis, are well-documented; less attention has historically been directed toward its potential to promote venous thromboembolism [3–5].

Pulmonary embolism arises when thrombus, typically originating in the deep veins of the lower limbs or pelvis, occludes the pulmonary arterial bed. It remains a major cause of preventable hospital mortality, and its management is guided substantially by the identification of precipitating factors — recent surgery, prolonged immobility, active cancer, thrombophilia, and pregnancy, among others [6]. Yet a proportion of PE cases occur in the apparent absence of such conventional triggers, raising the possibility of unrecognized procoagulant conditions. OSAS fits this profile on mechanistic grounds: sustained intermittent hypoxia has been proposed to activate nuclear factor-κB and hypoxia-inducible factor-1α, potentially promoting endothelial dysfunction, platelet aggregation, and impaired fibrinolysis, while sympathoadrenal surges may further compound the prothrombotic milieu [7–9]. However, these mechanisms were not directly measured in the present study and should be regarded as biologically plausible hypotheses rather than demonstrated explanations.

Clinical evidence linking OSAS to venous thromboembolism has been accumulating. Population-based cohort analyses have found an independent association between sleep-disordered breathing and incident PE [10], and several cross-sectional studies have observed strikingly high OSAS frequencies in PE survivors [11–14]. Nevertheless, most of this evidence derives from retrospective or questionnaire-based designs that lack objective polysomnographic validation. The present study was designed to fill this methodological gap by prospectively applying validated portable polysomnography (WatchPAT 200^®^) to a consecutive series of patients with radiologically confirmed PE, with the aims of determining OSAS prevalence and characterizing its clinical correlates in this population.

## Methods

### Study design and participants

This study was designed as a prospective evaluation of a patient cohort systematically followed and documented at the Department of Chest Diseases, İzmir Dr. Suat Seren Chest Diseases and Thoracic Surgery Training and Research Hospital, University of Health Sciences, İzmir, Türkiye, between 2010 and 2013. Patient follow-up, clinical parameters, and sleep studies were prospectively recorded during this period using standardized monitoring forms; formal institutional ethical approval for the subsequent compilation and statistical analysis of the accumulated dataset was granted by the Institutional Ethics Committee in 2016 (Approval No: 21/10/2016 / E5236–00032098799). All participants had previously provided written informed consent during their clinical follow-up for their clinical data to be evaluated and utilized for scientific research purposes. The study was conducted in strict accordance with the principles of the Declaration of Helsinki.

Consecutive adults (≥ 18 years) with a new, radiologically confirmed diagnosis of PE were eligible for inclusion. Exclusion criteria were refusal to participate and a prior diagnosis of OSAS or a previous polysomnographic study, so that the sleep assessment reflected a naïve baseline. Of the patients initially enrolled, five were excluded owing to technically inadequate WatchPAT recordings, leaving 45 patients in the final analysis.

### Radiological diagnosis

PE was confirmed by thoracic computed tomography pulmonary angiography (CTPA; Siemens multislice scanner, 1.5 mm slice thickness, intravenous iodinated contrast 400–450 mg iodine/kg at 3–5 mL/s) and/or ventilation/perfusion (V/Q) scintigraphy using Technetium-99 m macroaggregated albumin for perfusion and Technegas™ for ventilation, with PE identified by mismatched perfusion defects. Thirty-seven patients (82.2%) were diagnosed by CTPA and nine (20.0%) by V/Q scintigraphy, including one patient who underwent both modalities.

### Polysomnographic assessment

All participants underwent portable polysomnography using the WatchPAT 200^®^ device (Itamar Medical, Israel) within two weeks of PE diagnosis, either during inpatient stay or at home after discharge. WatchPAT records peripheral arterial tonometry, pulse oximetry, heart rate, and actigraphy; its diagnostic agreement with laboratory polysomnography is well-established [15, 16]. Recordings were scored by a sleep medicine specialist according to 2016 AASM criteria [17]. OSAS was defined as AHI ≥ 5 events/hour and classified as mild (5–14), moderate (15–29), or severe (≥ 30).

### Clinical and laboratory evaluation

All patients underwent structured clinical assessment on admission: measurement of blood pressure and peripheral oxygen saturation; thromboembolic risk stratification by the Wells score [18]; and transthoracic echocardiography for systolic pulmonary artery pressure (SPAP) and left ventricular ejection fraction. BMI was calculated as weight/height². OSAS-related symptoms (snoring, witnessed apnea, excessive daytime sleepiness) were elicited by standardized questioning. Laboratory investigations included complete blood count, serum biochemistry, D-dimer (Vidas-3 immunoassay), and cardiac biomarkers (CK-MB, troponin, NT-proBNP).

### Statistical analysis

Analyses were performed using SPSS version 22.0 (IBM Corp., Armonk, NY). Normality was assessed by the Shapiro–Wilk test. Continuous variables are expressed as median (interquartile range) and compared by the Mann–Whitney U test. Categorical variables are presented as n (%) and compared by Pearson chi-square or Fisher’s exact test. Correlations with AHI were assessed by Spearman’s rank coefficient. A two-tailed *p* < 0.05 was considered statistically significant.

## Results

### Patient characteristics and OSAS prevalence

**Fig. 1:**
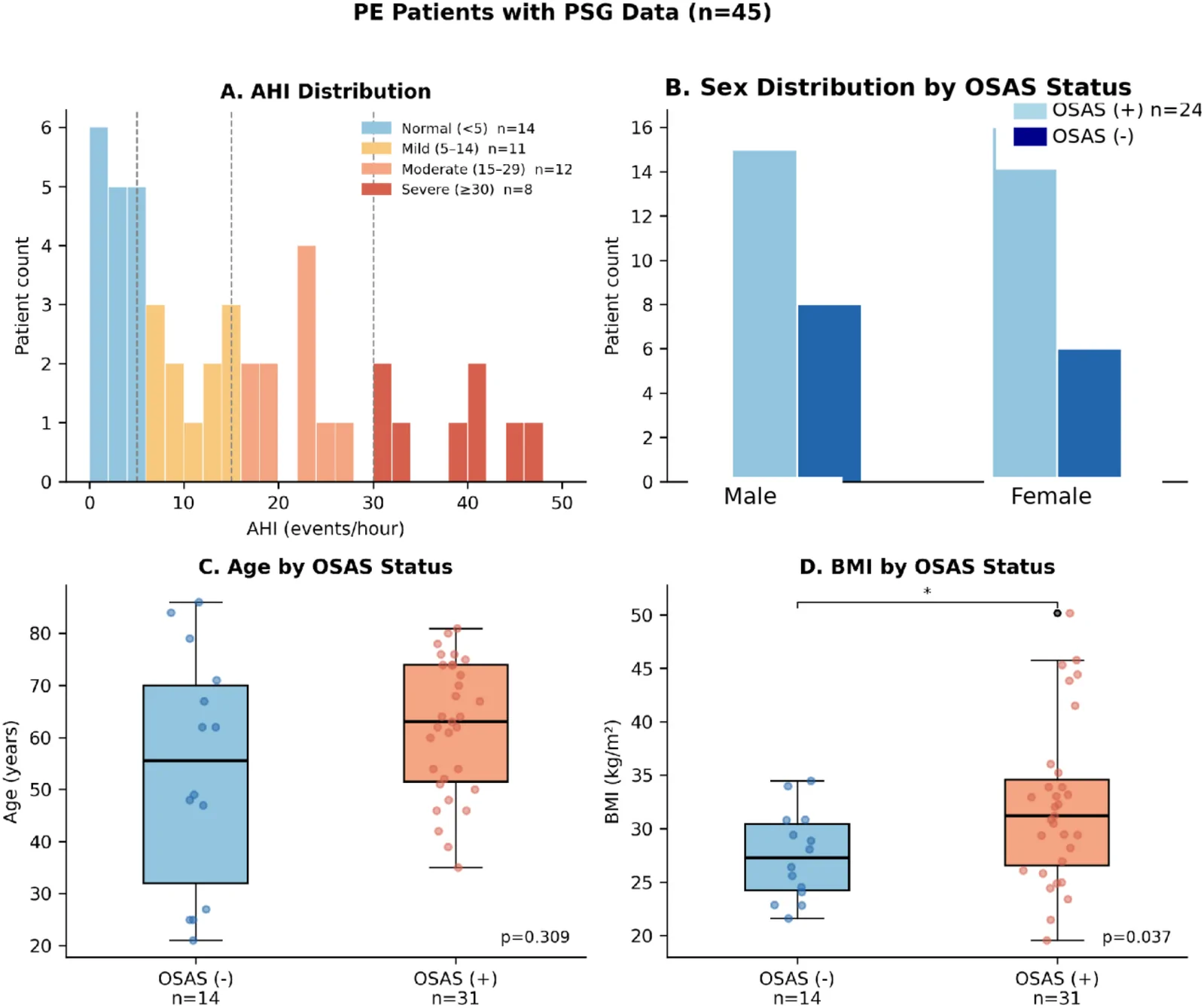
(**A**) Distribution of AHI values with severity-based shading. (**B**) Sex distribution by OSAS status. (**C**) Age and (**D**) BMI stratified by OSAS status. Boxes show median and IQR; dots represent individual patients. *p < 0.05 by Mann–Whitney U test. BMI: body mass index; IQR: interquartile range; OSAS: obstructive sleep apnea syndrome

The cohort comprised 45 patients (23 male, 22 female) with a median age of 62 years (IQR 48–74). OSAS was present in 31 patients (68.9%): 11 had mild, 12 moderate, and 8 severe disease. Figure 1 presents an overview of the cohort structure, including the AHI severity distribution and key anthropometric comparisons by OSAS status.

Baseline characteristics are presented in Table 1. Age did not differ significantly between OSAS-positive and OSAS-negative patients (medians 63.0 vs. 55.5 years; *p* = 0.309), nor did sex distribution (*p* = 0.824). BMI was significantly higher in the OSAS group [31.2 (26.5–34.6) vs. 27.3 (24.2–30.4) kg/m²; *p* = 0.037]. Among comorbidities, diabetes mellitus was numerically more frequent in OSAS-positive patients (38.7% vs. 7.1%; *p* = 0.071), a trend that fell short of significance, likely reflecting limited sample size. No other comorbidity differed significantly between groups. Importantly, Wells criteria frequencies were similar in both groups, and 38.7% of OSAS-positive patients had no identifiable Wells risk factor for PE (Table 1). Classical OSAS symptoms — snoring, witnessed apneas, excessive daytime sleepiness — were reported by fewer than one in four OSAS-positive patients and did not differ between groups, underscoring the subclinical nature of OSAS in this cohort.

All patients were haemodynamically stable at the time of sleep assessment. No patient required ICU admission or thrombolytic therapy, and echocardiography demonstrated normal right ventricular function in all cases, consistent with a low-to-intermediate risk PE cohort.

**Table 1:** Baseline characteristics, comorbidities, Wells criteria, and OSAS symptoms by OSAS status

Variable	All (*n* = 45)	OSAS− (*n* = 14)	OSAS+ (*n* = 31)	*p*
Demographics				
Age, years	62 (48–74)	55.5 (32.0–70.0)	63.0 (51.5–74.0)	0.309
Male sex, n (%)	23 (51.1%)	8 (57.1%)	15 (48.4%)	0.824
BMI, kg/m²	29.5 (25.6–33.9)	27.3 (24.2–30.4)	31.2 (26.5–34.6)	**0.037**
***Comorbidities***				
Hypertension	17 (37.8%)	3 (21.4%)	14 (45.2%)	0.235
Diabetes mellitus	13 (28.9%)	1 (7.1%)	12 (38.7%)	0.071
COPD	6 (13.3%)	1 (7.1%)	5 (16.1%)	0.728
Congestive heart failure	8 (17.8%)	1 (7.1%)	7 (22.6%)	0.405
Coronary artery disease	6 (13.3%)	3 (21.4%)	3 (9.7%)	0.549
Prior pulmonary embolism	2 (4.4%)	0 (0.0%)	2 (6.5%)	0.849
***Wells Criteria***				
DVT signs/symptoms	15 (33.3%)	4 (28.6%)	11 (35.5%)	0.909
Tachycardia (HR > 100 bpm)	6 (13.3%)	2 (14.3%)	4 (12.9%)	1.000
Immobilization (≥ 3 days)	15 (33.3%)	6 (42.9%)	9 (29.0%)	0.569
Recent surgery (< 4 weeks)	8 (17.8%)	2 (14.3%)	6 (19.4%)	1.000
Hemoptysis	3 (6.7%)	0 (0.0%)	3 (9.7%)	0.576
No Wells criterion identified	17 (37.8%)	5 (35.7%)	12 (38.7%)	1.000
***OSAS Symptoms***				
Snoring	8 (17.8%)	1 (7.1%)	7 (22.6%)	0.405
Witnessed apnea	1 (2.2%)	0 (0.0%)	1 (3.2%)	1.000
Excessive daytime sleepiness	6 (13.3%)	1 (7.1%)	5 (16.1%)	0.728

Data: median (IQR) for continuous variables, n (%) for categorical. Mann–Whitney U test (continuous); chi-square or Fisher’s exact test (categorical). Bold p-values: *p* < 0.05. BMI: body mass index; COPD: chronic obstructive pulmonary disease; IQR: interquartile range; OSAS: obstructive sleep apnea syndrome.

### Laboratory and clinical findings

**Fig. 2:**
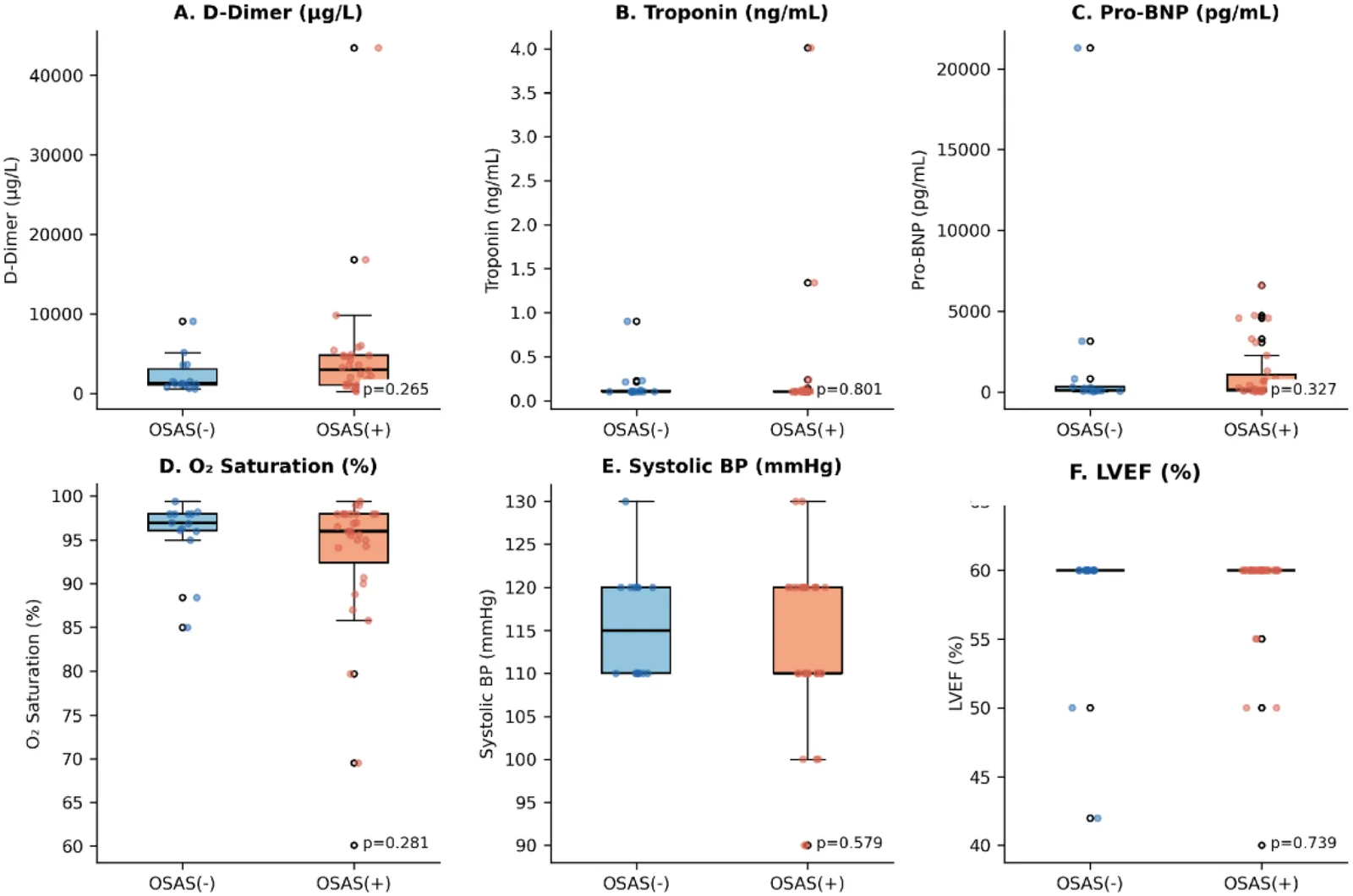
Box-and-whisker plots with individual data points overlaid. Bold asterisk (*) denotes p < 0.05 by Mann–Whitney U test; NT-proBNP: N-terminal pro-brain natriuretic peptide; OSAS: obstructive sleep apnea syndrome; LVEF: Left Ventricular Ejection Fraction; BP: blood pressure

Laboratory and admission clinical data are summarized in Table 2 and illustrated in Fig. 2. BUN was significantly higher in OSAS-positive patients [36.0 (25.1–52.0) vs. 19.5 (16.2–43.5) mg/dL; *p* = 0.026]. All other laboratory parameters, including D-dimer, troponin, NT-proBNP, hemoglobin, and creatinine, were similar between groups. Daytime oxygen saturation (*p* = 0.281), systolic blood pressure (*p* = 0.579), diastolic blood pressure (*p* = 0.750), and echocardiographic LVEF (*p* = 0.739) did not differ significantly.

**Table 2:** Laboratory values and admission clinical measurements by OSAS status

Parameter	OSAS− (*n* = 14)	OSAS+ (*n* = 31)	*p*
Laboratory Values			
Hemoglobin, g/dL	12.7 (11.1–14.0)	12.5 (11.2–13.6)	0.768
Hematocrit, %	38.0 (33.3–42.0)	37.0 (33.5–41.3)	0.787
BUN, mg/dL	19.5 (16.2–43.5)	36.0 (25.1–52.0)	**0.026**
Creatinine, mg/dL	0.9 (0.8–1.0)	0.9 (0.8–1.2)	0.333
D-Dimer, µg/L	1254 (1054–3072)	2927 (1021–4814)	0.265
Troponin, ng/mL	0.10 (0.10–0.11)	0.10 (0.10–0.11)	0.801
NT-proBNP, pg/mL	80 (51–298)	127 (77–1065)	0.327
***Admission Clinical Parameters***			
Daytime SpO₂, %	97.0 (96.0–98.0)	96.0 (92.4–98.0)	0.281
Systolic BP, mmHg	115 (110–120)	110 (110–120)	0.579
Diastolic BP, mmHg	70 (70–70)	70 (70–80)	0.750
Echocardiographic LVEF, %	60 (60–60)	60 (60–60)	0.739

Data: median (IQR). Mann–Whitney U test. Bold p-values: p < 0.05. AHI: apnea–hypopnea index; IQR: interquartile range; ODI: oxygen desaturation index; OSAS: obstructive sleep apnea syndrome; SpO₂: peripheral oxygen saturation

### Polysomnographic findings

**Fig. 3:**
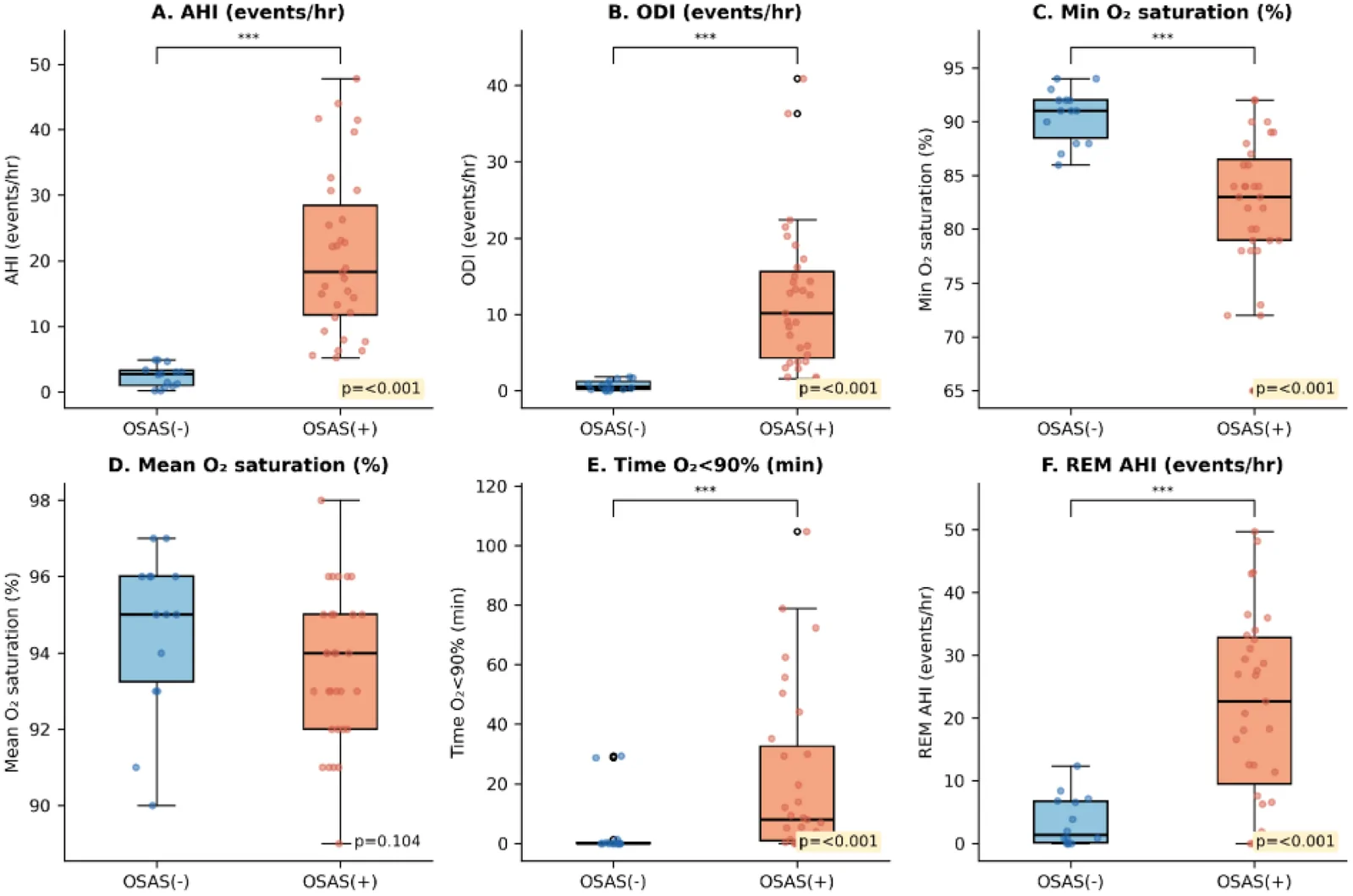
WatchPAT-derived polysomnographic parameters by OSAS status. Box-and-whisker plots with individual patient data overlaid. Highlighted p-values indicate significance (p < 0.05, Mann–Whitney U). AHI: apnea–hypopnea index; ODI: oxygen desaturation index; OSAS: obstructive sleep apnea syndrome; SpO₂: peripheral oxygen saturation

PSG parameters are shown in Table 3; Fig. 3. All indices of respiratory event frequency and nocturnal hypoxemic burden were substantially higher in OSAS-positive patients. Total AHI [18.3 (11.8–28.5) vs. 2.8 (1.0–3.3) events/hour; *p* < 0.001], REM AHI [22.7 (9.5–32.9) vs. 1.4 (0.2–6.8); *p* < 0.001], NREM AHI [16.3 (9.1–24.6) vs. 2.0 (1.1–3.3); *p* < 0.001], and ODI [10.2 (4.3–15.6) vs. 0.5 (0.2–1.2) events/hour; *p* < 0.001] all differed markedly. Minimum nocturnal SpO₂ was significantly lower in OSAS patients [83.0% (79.0–86.5) vs. 91.0% (88.5–92.0); *p* < 0.001], and the time spent with SpO₂ <90% was considerably longer [8.0 (1.0–32.7) vs. 0.0 (0.0–0.3) minutes; *p* = 0.001]. Mean nocturnal SpO₂, while numerically lower in OSAS patients (94.0% vs. 95.0%), did not reach statistical significance (*p* = 0.104). Nocturnal heart rate parameters were similar between groups.

**Table 3:** WatchPAT-derived polysomnographic parameters by OSAS status

Parameter	OSAS− (*n* = 14)	OSAS+ (*n* = 31)	*p*
AHI (events/hour)			
AHI total	2.8 (1.0–3.3)	18.3 (11.8–28.5)	**< 0.001**
AHI REM	1.4 (0.2–6.8)	22.7 (9.5–32.9)	**< 0.001**
AHI NREM	2.0 (1.1–3.3)	16.3 (9.1–24.6)	**< 0.001**
Body-position AHI	2.4 (1.0–3.9)	17.6 (8.2–33.5)	**< 0.001**
***Nocturnal Oxygenation***			
ODI, events/hour	0.5 (0.2–1.2)	10.2 (4.3–15.6)	**< 0.001**
Mean nocturnal SpO₂, %	95.0 (93.2–96.0)	94.0 (92.0–95.0)	0.104
Minimum nocturnal SpO₂, %	91.0 (88.5–92.0)	83.0 (79.0–86.5)	**< 0.001**
Time with SpO₂ <90%, min	0.0 (0.0–0.3)	8.0 (1.0–32.7)	**0.001**
% sleep time SpO₂ <90%	0.0 (0.0–0.2)	1.8 (0.3–10.5)	**0.001**
***Nocturnal Heart Rate***			
Mean heart rate, bpm	69.0 (65.2–76.5)	74.0 (68.0–84.5)	0.215
Minimum heart rate, bpm	54.0 (50.5–62.8)	50.0 (43.0–58.0)	0.188
Data: median (IQR). Mann–Whitney U test. Bold p-values: p < 0.05. AHI: apnea–hypopnea index; IQR: interquartile range; ODI: oxygen desaturation index; OSAS: obstructive sleep apnea syndrome; SpO₂: peripheral oxygen saturation			

### Correlations with AHI

**Fig. 4:**
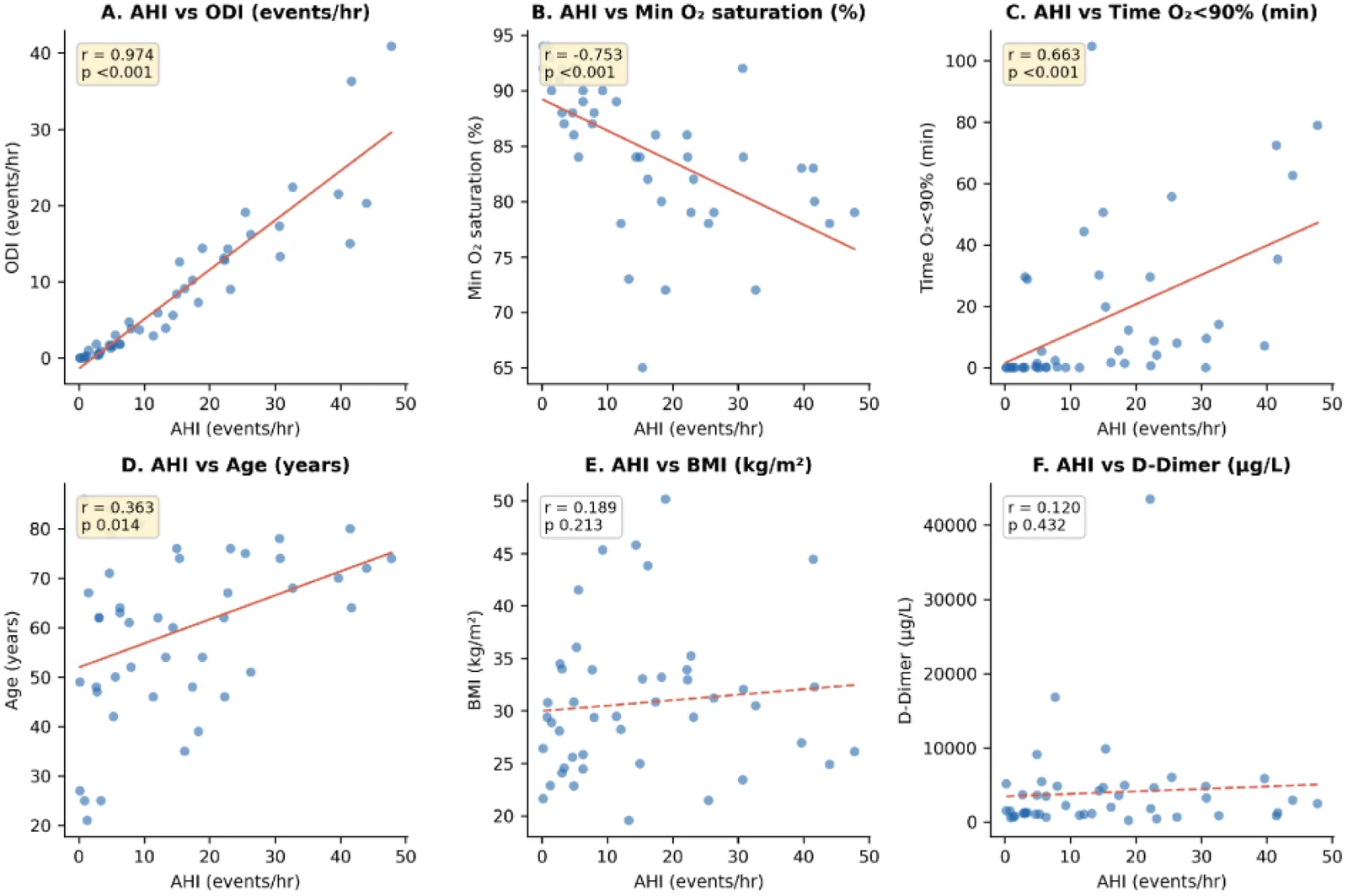
Each panel shows individual patient data points with a fitted trend line (solid = p < 0.05; dashed = NS). Correlation coefficient (r) and p-value are displayed within each panel. AHI: apnea–hypopnea index; BMI: body mass index; ODI: oxygen desaturation index; SpO₂: peripheral oxygen saturation

Spearman correlations between AHI and key clinical parameters are presented in Table 4 and illustrated in Fig. 4. AHI showed the strongest association with ODI (*r* = 0.974; *p* < 0.001) — an expected, near-perfect correlation reflecting that both indices measure oxygen-event frequency — followed by minimum nocturnal SpO₂ (*r* =− 0.753; *p* < 0.001), time with SpO₂ <90% (*r* = 0.663; *p* < 0.001), and mean nocturnal SpO₂ (*r* =− 0.430; *p* = 0.003). AHI also correlated positively with age (*r* = 0.363; *p* = 0.014). No significant correlations were observed with D-dimer, troponin, NT-proBNP, or hemodynamic parameters.

**Table 4:** Spearman rank correlations between AHI and clinical and polysomnographic parameters

Variable	Spearman *r*	*p*-value	Significant
PSG parameters			
ODI (events/hour)	**0.974**	**< 0.001**	**Yes**
Minimum nocturnal SpO₂ (%)	**−0.753**	**< 0.001**	**Yes**
Time with SpO₂ <90% (min)	**0.663**	**< 0.001**	**Yes**
Mean nocturnal SpO₂ (%)	**−0.430**	**0.003**	**Yes**
***Demographics & Anthropometrics***			
Age (years)	**0.363**	**0.014**	**Yes**
BMI (kg/m²)	0.189	0.213	No
***Laboratory***			
D-Dimer (µg/L)	0.120	0.432	No
Troponin (ng/mL)	−0.053	0.730	No
NT-proBNP (pg/mL)	0.174	0.253	No
***Hemodynamics***			
Daytime SpO₂ (%)	−0.169	0.268	No
Systolic BP (mmHg)	−0.290	0.054	No

Bold values: p < 0.05. AHI: apnea–hypopnea index; BMI: body mass index; BP: blood pressure; ODI: oxygen desaturation index; SpO₂: peripheral oxygen saturation.

### OSAS severity and risk factor distribution

**Fig. 5:**
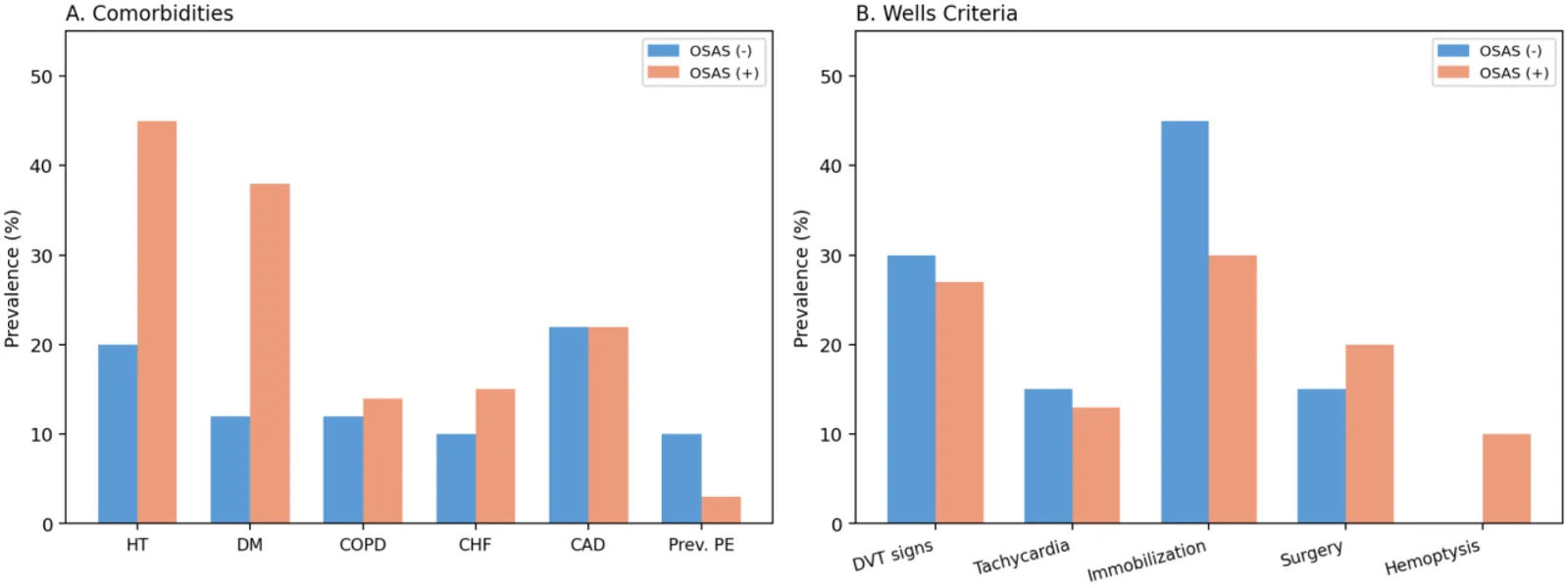
Bar heights represent percentage of patients in each group. None of the between-group differences reached statistical significance. CAD: coronary artery disease; CHF: congestive heart failure; COPD: chronic obstructive pulmonary disease; DM: diabetes mellitus; DVT: deep vein thrombosis; HT: hypertension; OSAS: obstructive sleep apnea syndrome; PE: pulmonary embolism

Figure 5 compares comorbidity burden and Wells criterion frequencies across OSAS and non-OSAS groups. Despite a numerical trend for higher diabetes prevalence and hypertension in the OSAS group, none of these categorical differences reached conventional significance. The distribution of Wells criteria was also similar between groups, suggesting that the two groups were comparable with respect to these specific conventional risk factors, although this does not exclude the presence of other thromboembolic risk factors not captured by the Wells scoring system.

**Fig. 6:**
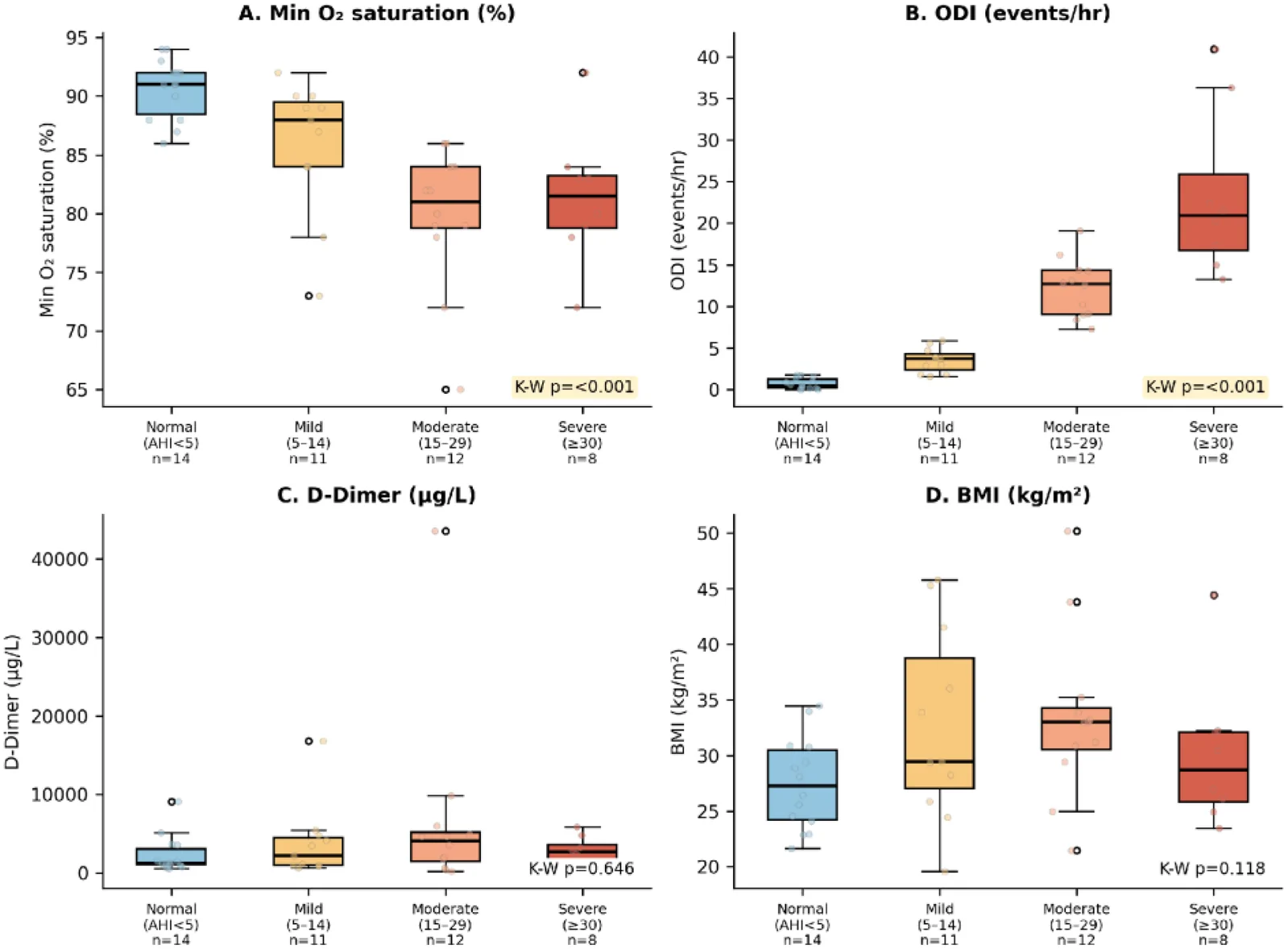
Box-and-whisker plots across four AHI severity categories. Kruskal–Wallis p-values are shown. A clear gradient of worsening oxygenation with increasing OSAS severity is evident. AHI: apnea–hypopnea index; BMI: body mass index; ODI: oxygen desaturation index; OSAS: obstructive sleep apnea syndrome; SpO₂: peripheral oxygen saturation

Figure 6 displays key PSG and clinical parameters stratified across all four OSAS severity categories (normal, mild, moderate, severe), showing a progressive worsening of nocturnal hypoxemic burden with increasing OSAS severity.

## Discussion

The main finding of this prospective study is a high prevalence of OSAS — 68.9% — in patients with newly diagnosed, radiologically confirmed PE. This substantially exceeds the estimated background prevalence of OSAS in middle-aged adults (approximately 9–13%) and is consistent with the upper range of estimates from prior studies in PE populations [11–14]. The study adds to this literature by relying on validated objective polysomnography rather than questionnaire-based screening, and by enrolling patients prospectively within a defined window after PE diagnosis.

One of the most noteworthy observations was that nearly 40% of OSAS-positive patients had no identifiable Wells risk factor for PE, a proportion virtually identical to that observed in the OSAS-negative group. This finding suggests that the high prevalence of OSAS in our cohort cannot be readily attributed to an excess burden of conventional PE risk factors. While causality cannot be inferred from the present study, the marked nocturnal hypoxemic burden observed in OSAS-positive patients is consistent with biological mechanisms previously implicated in thrombotic susceptibility. The nocturnal hypoxemia data support this interpretation. Minimum nocturnal SpO₂ was markedly lower in OSAS patients (83.0% vs. 91.0%; *p* < 0.001), ODI was greatly elevated, and the time spent below 90% saturation was substantially prolonged. The strong negative correlation between AHI and minimum nocturnal SpO₂ (*r* = − 0.753; *p* < 0.001) directly quantifies this relationship and aligns mechanistically with evidence that hypoxia-inducible factor activation promotes a procoagulant phenotype in endothelial cells [7, 9].

The significantly higher BMI in OSAS-positive patients (31.2 vs. 27.3 kg/m²; *p* = 0.037) was expected given the well-established relationship between obesity and upper airway collapsibility [19]. Obesity also promotes venous stasis through raised intra-abdominal pressure and reduced physical activity. Although obesity may contribute to both OSAS and thromboembolic risk, the current study was not designed to determine the relative contribution of obesity, nocturnal hypoxemia, or other metabolic factors to the observed association.

The significantly elevated BUN in OSAS patients (*p* = 0.026) is an intriguing finding. In OSA, recurrent sympathoadrenal activation and renal arterial vasoconstriction can impair renal perfusion over time, raising serum urea independently of frank renal disease [20]. This has been observed in other series, and in our cohort it may reflect the cumulative hemodynamic consequence of years of untreated sleep-disordered breathing. The trend toward higher diabetes prevalence in OSAS patients (38.7% vs. 7.1%; *p* = 0.071), though not statistically significant, is consistent with the well-documented bidirectional relationship between OSAS and insulin resistance and warrants examination in larger cohorts.

Perhaps the most clinically pressing implication of our findings is the low rate of classical OSAS symptoms in OSAS-positive patients. Fewer than one in four OSAS-positive patients reported snoring, witnessed apneas, or excessive daytime sleepiness, and none of these symptom frequencies differed from the OSAS-negative group. Symptom-based screening would therefore miss the majority of OSAS cases in this population. Given that CPAP therapy reduces markers of endothelial dysfunction, platelet activation, and sympathoadrenal tone in OSAS [21], diagnosing and treating OSAS in PE patients may reduce the risk of recurrent thromboembolism — a question that deserves formal prospective evaluation.

Cardiac biomarkers (troponin, NT-proBNP) and D-dimer did not differ between groups. This is consistent with other studies and suggests that the prothrombotic influence of OSAS operates through chronic subclinical changes rather than acute hemodynamic derangements detectable at the time of PE presentation. AHI did not correlate with systolic blood pressure, LVEF, or D-dimer, reinforcing the view that the OSAS–PE link is mediated primarily through the nocturnal hypoxemic pathway rather than acute hemodynamic effects.

Several limitations deserve acknowledgment. The modest sample size of 45 patients, including only 14 in the OSAS-negative group, substantially restricts statistical power and increases the risk of type II error, particularly for secondary comparisons where true differences may exist but remain undetected. Furthermore, due to the limited sample size (*n* = 45) and the particularly small number of patients in the OSAS-negative group (*n* = 14), multivariable logistic regression analysis was not performed, as this would risk statistical overfitting. Consequently, while potential confounding by obesity, diabetes mellitus, and other metabolic comorbidities cannot be completely excluded, larger cohorts are warranted in future studies to clarify the independent contribution of these factors. The single-center design and study period (2010–2013) may limit generalizability. Although WatchPAT lacks EEG-based sleep staging, direct airflow measurement, and comprehensive respiratory effort monitoring, and although peripheral arterial tone signals may theoretically be influenced by haemodynamic instability or sympathetic activation in acutely ill patients, this potential bias was mitigated in our cohort. All included patients were haemodynamically stable, non-massive cases managed in the general ward without clinical or echocardiographic signs of right ventricular dysfunction. Furthermore, conducting sleep assessments within a defined two-week window rather than the hyper-acute phase allowed patients to reach a steady clinical baseline, thereby supporting the validity of the PAT signals and subsequent AHI estimations. Nonetheless, WatchPAT is an FDA-approved device that has been extensively validated against laboratory polysomnography [15, 16] and is recognized by the American Academy of Sleep Medicine as an acceptable diagnostic tool [17]. Furthermore, as all patients in the present study were evaluated using the same device under identical conditions, any systematic measurement bias would have affected both groups equally, preserving the validity of between-group comparisons. Prospective multicenter studies with longer follow-up are needed to establish whether OSAS treatment modifies PE recurrence risk.

## Conclusions

OSAS was identified in approximately 70% of patients with newly diagnosed PE in this prospective cohort—a prevalence substantially higher than that reported in the general population. OSAS-positive patients exhibited significantly higher BMI, elevated BUN levels, and a markedly greater nocturnal hypoxemic burden, characterized by lower minimum nocturnal SpO₂, higher ODI values, and prolonged time spent below 90% oxygen saturation. Despite these differences, the frequency of classical Wells criteria was similar between groups, and a substantial proportion of OSAS-positive patients had no identifiable conventional precipitating factor for PE. These findings suggest that the high prevalence of OSAS in this cohort cannot be fully explained by differences in established PE risk factors alone and are consistent with previously proposed biological links between sleep-disordered breathing and thrombotic susceptibility. The predominantly asymptomatic nature of OSAS in this population indicates that reliance on clinical symptoms alone may fail to identify a considerable proportion of affected patients. Further prospective studies incorporating larger populations, matched control groups, and multivariable analyses are needed to clarify the nature of the observed association and to evaluate the clinical utility and cost-effectiveness of targeted OSAS screening strategies in PE patients, particularly in those without conventional risk factors.

## Data Availability

The datasets generated and analysed during the current study are not publicly available due to patient privacy and confidentiality restrictions in accordance with the ethical approval granted by the Institutional Ethics Committee of the University of Health Sciences, İzmir Dr. Suat Seren Chest Diseases and Thoracic Surgery Training and Research Hospital (Approval No: 21/10/2016 / E5236–00032098799), but are available from the corresponding author (M.C.) upon reasonable request.
